# Association between prothrombin time-international normalized ratio and prognosis of post-cardiac arrest patients: A retrospective cohort study

**DOI:** 10.3389/fpubh.2023.1112623

**Published:** 2023-01-20

**Authors:** Yiyang Tang, Jing Sun, Zaixin Yu, Benhui Liang, Baohua Peng, Jing Ma, Xiaofang Zeng, Yilu Feng, Qin Chen, Lihuang Zha

**Affiliations:** ^1^Department of Cardiology, Xiangya Hospital, Central South University, Changsha, Hunan, China; ^2^National Clinical Research Center for Geriatric Disorders (Xiang Ya), Changsha, Hunan, China

**Keywords:** international normalized ratio, cardiac arrest, critical care, all-cause mortality, MIMIC-IV database

## Abstract

**Background:**

Cardiac arrest (CA) can activate blood coagulation. This study aimed to explore the potential prognostic value of prothrombin time–international normalized ratio (INR) in post-CA patients.

**Methods:**

The clinical data of eligible subjects diagnosed with CA was extracted from the MIMIC-IV database as the training cohort. Restricted cubic spline (RCS), Kaplan–Meier (K-M) survival curve, and Cox regression analyses were conducted to elucidate the association between the INR and all-cause mortality of post-CA patients. Subgroup analysis, propensity score matching (PSM), and inverse probability of treatment (IPTW) were also conducted to improve stability and reliability. Data of the validation cohort were collected from the eICU database, and logistic-regression analyses were performed to verify the findings of the training cohort.

**Results:**

A total of 1,324 subjects were included in the training cohort. A linear correlation existed between INR and the risk of all-cause death of post-CA patients, as shown in RCS analysis, with a hazard ratio (HR) >1 when INR exceeded 1.2. K-M survival curve preliminarily indicated that subjects with INR ≥ 1.2 presented lower survival rate and shorter survival time, and the high level of INR was independently associated with 30-day, 90-day, 1-year, and in-hospital mortalities, with multivariate-adjusted HR of 1.44 (1.20, 1.73), 1.46 (1.23, 1.74), 1.44 (1.23, 1.69), and 1.37 (1.14, 1.64), respectively. These findings were consistent and robust across the subgroup analysis, PSM and IPTW analyses, and validation cohort.

**Conclusions:**

We systematically and comprehensively demonstrated that elevated INR was associated with increased short- and long-term all-cause mortality of post-CA patients. Therefore, elevated INR may be a promising biomarker with prognosis significance.

## 1. Introduction

Cardiac arrest (CA) is defined as the sudden cessation of cardiac ejection for various reasons. It has the characteristics of interrupted systemic circulation, respiratory arrest, and loss of consciousness (1). The incidence of CA is not uncommon, with approximately 140.7 out-of-hospital CA per 100,000 individuals in the United States, compared with 17.16 in-hospital CA per 1,000 hospitalizations (2). The treatment and care for CA patients have made considerable progress in recent years, but the prognosis of this group of patients remains poor, with an in-hospital survival rate of only 28.7% (3, 4). Clinicians need to deeply study the pathophysiological mechanism of the occurrence and progression of CA to search for new therapeutic targets. They also need to identify and determine some novel biomarkers related to the prognosis of post-CA patients to stratify high-risk patients promptly and take more effective therapeutic measures. All these endeavors can help improve the prognosis of patients (5).

Abnormity in the coagulation–fibrinolysis system is an important pathophysiological feature of post-CA patients (4, 6, 7). During CA and resuscitation, hypoxia and acidosis often occur. They can inflict vascular endothelial-cell damage, thereby stimulating tissue-factor release and thus initiating the exogenous-coagulation process. Besides, the excessively activated inflammatory response induced by the release of injury-related molecular patterns after tissue and cell damage can accelerate the activation of tissue-factor-dependent coagulation and also promote coagulation factor XII- and XI-dependent blood coagulation (8). Meanwhile, the endogenous fibrinolytic system is partially suppressed with decreased antithrombin, tissue-factor pathway inhibitor, protein C/S, and other anticoagulant substances (9). Coagulation and fibrinolysis-related indicators including activated partial thromboplastin time (APTT), fibrinogen degradation products, and D-dimer have been found to be closely related to the prognosis of post-CA patients and are expected to be promising biomarkers with prognosis significance (10–12).

As a sensitive and reliable indicator for screening and assessing exogenous-coagulation system disorders, the prothrombin time-international normalized ratio (INR) is extensively used to monitor anticoagulation therapy, assess liver dysfunction, and evaluate coagulation abnormalities such as DIC (13). A series of studies has also shown that elevated INR is closely associated with an adverse prognosis in various kinds of diseases, including trauma (14), sepsis (15), cerebral hemorrhage (16), and acute decompensated heart failure (17), with a promising application. However, the relationship between INR and the prognosis of post-CA patients remains unclear, particularly in long-term all-cause mortality. Accordingly, the present study aimed to illustrate the relationship between INR and short- and long-term all-cause mortalities in post-CA patients and thus identify a simple, objective, and reliable prognostic indicator. Our results can serve as a reference for the clinical management of post-CA patients.

## 2. Materials and methods

### 2.1. Data sources

The present research was a retrospective cohort study. All subjects' data were extracted from two large critical-care medical databases, which are free and open to researchers from all over the world. Data of the training cohort were collected from the Multiparameter Intelligent Monitoring in Intensive Care IV (MIMIC-IV; version 2.0) database (18, 19), which is jointly developed and run by the Massachusetts Institute of Technology (Cambridge, MA, USA) and the Beth Israel Deaconess Medical Center (BIDM, Boston, MA, USA). This database contains the detailed and comprehensive clinical data of about 250,000 patients admitted to the intensive care unit (ICU) and emergency unit of the Beth Israel Deaconess Medical Center from 2008 to 2019, including demography, laboratory tests, documented vital signs, medications administered, and so on. In-hospital and out-of-hospital death information is also available. The longest follow-up period for each patient was 1 year after their last discharge, providing great data support for clinical studies.

Data of the validation cohort were extracted from the eICU collaborative research database (20, 21), which is a multicenter database containing data of more than 200,000 ICU admissions across 208 United States hospitals between 2014 and 2015. Funded by the Philips eICU program, this database also includes vital signs, laboratory measurements, severity of illness, and diagnosis and treatment information. However, survival data and out-of-hospital follow-up information are unavailable.

### 2.2. Statement and authorization

The MIMIC-IV and eICU databases were de-identified, and patient identifiers were removed according to the Health Insurance Portability and Accountability Act Safe Harbor provision. The database was approved by ethical review boards, and requesting another ethical review for the present study was unnecessary. According to the database protocol, the author Tang passed the exam for “Protecting Human Research Participants” and gained access to the MIMIC-IV database (Record ID: 43449634). The reporting specifications for this study were in compliance with the STROBE statement.

### 2.3. Study population

Patients diagnosed with CA based on the International Classification of Diseases versions 9 and 10 diagnosis codes (ICD-9&10, “4,275,” “I46,” “I,462,” “I,468,” and “I,469”) were included in this study. Further screening criteria for research subjects were as follows: (1) only the first admission for patients with multiple ICU admissions was considered; (2) adult patients were aged 18 years and above; (3) patients had an ICU stay of more than 24 h; (4) patients had a calculated survival time >0 (some organ-donation patients died earlier than the time of admission); (5) patients had no missing INR data within 24 h after ICU admission; and (6) patients were not treated with warfarin.

### 2.4. Research variable and outcomes

The independent variable was the INR of post-CA patients within 24 h after admission to ICU. The outcome events of interest were all-cause death (in-hospital or within 30-day, 90-day, and 1-year after admission).

### 2.5. Data extraction and processing

Using PostgreSQL software (version 9.6, https://www.postgresql.org/), the author Tang extracted the clinical data of all subjects after obtaining authorization. These data included demographic characteristics, vital signs, laboratory tests, comorbidities, severity of illness scores, and treatments administered. Demographic characteristics included age, gender, and race. Vital signs included heart rate, respiratory rate, mean blood pressure (MBP), temperature, and pulse oxygen saturation. Laboratory tests included hemoglobin, white blood cells (WBCs), platelets, hematocrit, anion gap, bicarbonate, calcium, chloride, sodium, potassium, serum creatinine, blood urea nitrogen (BUN), glucose, alanine transaminase (ALT), bilirubin, INR, and APTT, which were collected within 24 h upon admission to ICU. Comorbidities [hypertension, diabetes mellitus, heart failure, atrial fibrillation, acute myocardial infarction (AMI), valvular heart disease (VHD), cardiomyopathy, pulmonary embolism, pulmonary hypertension, chronic obstructive pulmonary disease, renal diseases, liver diseases, stroke, and malignant tumor] were identified by corresponding ICD-9&10 codes, and the Charlson comorbidity index was calculated. The severity of ill scores was recorded for each subject, including the Sequential Organ Failure Assessment (SOFA) score and Simplified Acute Physiology Score II (SAPSII). Treatment measures such as mechanical ventilation, vasopressors, renal replacement therapy (RRT), transfusion of fresh frozen plasma (FFP) were also extracted. Variables with more than 20% missing values were not included in subsequent analyses. Variables with fewer missing data were filled with multiple imputation using the “mice” package of the R program.

### 2.6. Statistical analyses

Data of continuous variables were presented as the mean (standard deviations) for normal distributions or median (interquartile range) for skewed distributions, whereas categorical variables were expressed as numbers of cases and percentages. Two-group comparisons (survivor vs. nonsurvivor group, low INR vs. high-INR group) were conducted with student's *t*-test, Mann–Whitney U test, and χ^2^ test (or Fisher's exact test) for normally distributed continuous, non-normally distributed continuous, and categorical variables, respectively.

Restricted cubic-spline analysis based on Cox proportional hazard model was performed to visualize the linear or nonlinear association between INR and all-cause mortality of post-CA patients, as well as to identify the inflection point as the cutoff to divide the whole cohort into low-INR and high-INR groups. Kaplan–Meier (K-M) survival curves were applied to visualize the cumulative probability of all-cause death across INR strata. Log-rank tests were used to compare the differences in risk between the groups.

Univariate and multivariate Cox proportional hazard models were conducted to assess the association between all-cause mortality and the two INR groups. They were presented as hazard ratios (HRs) and 95% confidence intervals (CI). No variables were adjusted in the univariate Cox regression analysis. The multivariate Cox model was adjusted with those variates whose effect on the independent variable exceeded 10% or clinically significant variables according to past experience, including age, gender, race, type of ICU for the first time, SOFA, SAPSII, Charlson comorbidity index, heart rate, MBP, anion gap, BUN, mechanical ventilation, vasopressors, RRT, aspirin, heparin, and the transfusion of FFP and platelets.

To further improve the reliability of the conclusion, the propensity score matching (PSM) and propensity score-based inverse probability of treatment (IPTW) were performed to balance the baseline characteristics of the two groups of subjects. In this study, nonparsimonious multivariable logistic-regression model was used to estimate the propensity score, and subjects in the low- and high-INR groups were matched one-to-one based on the propensity score by using the nearest neighbor matching algorithm with a caliper width of 0.25. For the IPTW analysis, a pseudo-population was generated using the estimated propensity scores as weights. The standardized mean difference was calculated to examine the efficiency of PSM and IPTW. For sensitivity analysis, K-M curves and Cox regression analysis were also reconducted on the PSM and IPTW cohorts to check the potential impact of INR on the all-cause mortality of patients with post-CA.

To evaluate the robustness of the findings of the present study, subgroup analysis was conducted to determine whether the association between INR and 1-year all-cause mortality of patients with post-CA was modified by age, gender, race, comorbidities, and disease severity. Sensitivity analyses were also performed in the post-CA patients with or without cardiac diseases (heart failure, AMI, cardiomyopathy, and VHD). The receiver operator characteristic (ROC) curve was drawn, and the area under the curve was calculated to compare the predictive performance of INR, SOFA, and SAPSII score in predicting the 1-year all-cause mortality of patients with post-CA.

The above statistical analyses were performed in R software (version 4.2.2) and EmpowerStats software (version 3.0, http://www.empowerstats.com/cn/, X&Y Solutions, Inc, Boston, MA, USA) for Windows system. p < 0.05 (two sided) was considered statistically significant.

## 3. Results

### 3.1. Clinical characteristics of subjects

**Figure 1:**
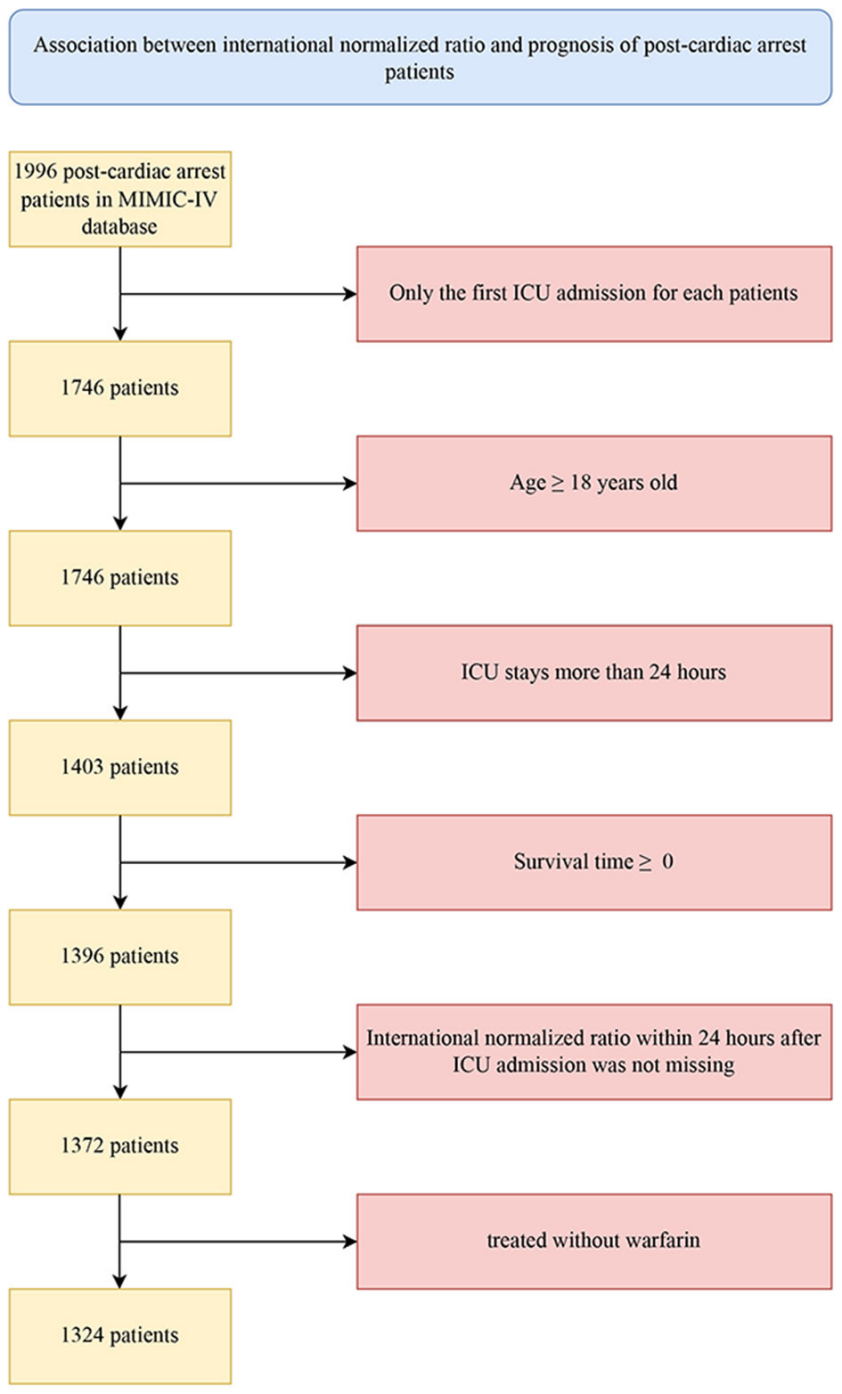
Flowchart for subjects' inclusion and exclusion from the MIMIC-IV database.

The procedure of participant enrollment in this study is illustrated in Figure 1. A total of 1,324 eligible patients diagnosed with CA were ultimately included in the training cohort. In general, the median age of patients was 67.33 years old, of whom 821 were male and 503 were female. White ethnicity was the majority, with a total of 759, accounting for 57.33% of the whole cohort. At 1-year after admission, 502 patients (37.92%) survived and 822 patients (62.08%) died of various causes. Table 1 demonstrates the clinical characteristics of the survivors and nonsurvivors at 1-year follow-up. Compared with the survivor group, patients in the nonsurvivor presented a higher level of INR, APTT, WBCs, ALT, BUN, creatinine, and anion gap but lower hemoglobin, bicarbonate, calcium, and chloridion levels. In terms of vital signs, nonsurvival patients tended to have lower MBP, temperature, weight, and urine amount. However, their heart rates and respiratory rate increased significantly. Patients in the nonsurvivor tended to be more serious, with greater SOFA and SAPSII scores. They had higher incidence of comorbidities, including diabetes mellitus, renal and liver diseases, stroke, and malignant tumors. Besides, the use of mechanical ventilation, RRT, and vasopressor, as well as the transfusion of FFP, was also more common in the nonsurvivor than the survivor group, whereas the nonsurvivors were less likely to receive aspirin and heparin.

**Table 1:** Comparisons of the baseline characteristics between survivors and non-survivors at 1-year follow-up in the training cohort.

**Variables**	**All**	**Survivors**	**Non-survivors**	***p* value**
N	1,324	502	822	
Age, years	67.33 (56.24–79.19)	64.22 (54.31–74.67)	69.64 (57.12–80.45)	<0.001
Male, %	821 (62.01%)	328 (65.34%)	493 (59.98%)	0.051
**Race, %**				0.027
White	759 (57.33%)	314 (62.55%)	445 (54.14%)	
Black	139 (10.50%)	48 (9.56%)	91 (11.07%)	
Asian	36 (2.72%)	12 (2.39%)	24 (2.92%)	
Other	390 (29.46%)	128 (25.50%)	262 (31.87%)	
**First care unit**				<0.001
CCU	532 (40.18%)	259 (51.59%)	273 (33.21%)	
MICU	474 (35.80%)	135 (26.89%)	339 (41.24%)	
Other	318 (24.02%)	108 (21.51%)	210 (25.55%)	
**Vital signs**				
HR, beats/minute	82.02 (70.80–95.29)	79.16 (69.23–90.33)	84.67 (72.52–98.00)	<0.001
RR, times/minute	19.90 (17.40–23.08)	19.18 (16.87–22.29)	20.50 (17.86–23.57)	<0.001
MBP, mmHg	77.28 (70.89–84.67)	78.49 (72.45–85.33)	76.61 (69.79–84.13)	0.003
Temperature, °C	36.72 (36.26–37.10)	36.78 (36.51–37.14)	36.66 (36.07–37.05)	<0.001
SpO2, %	97.82 (96.15–99.08)	97.83 (96.36–99.00)	97.82 (96.04–99.16)	0.734
Weight, kg	80.00 (67.00–95.40)	83.45 (70.05–99.25)	78.80 (65.02–92.67)	<0.001
Urine amount, L	1.26 (0.67–2.13)	1.59 (0.96–2.47)	1.09 (0.45–1.84)	<0.001
**Laboratory tests**				
Hemoglobin, g/dL	10.10 (8.40–12.20)	10.90 (9.00–12.78)	9.60 (8.03–11.60)	<0.001
Platelet, K/μl	163.00 (119.00–223.25)	162.50 (125.00–212.75)	163.50 (113.25–230.00)	0.843
WBC, K/μl	10.00 (7.10–13.60)	9.45 (7.00–12.90)	10.40 (7.10–14.20)	0.014
Hematocrit	30.90 (25.68–36.70)	32.90 (27.10–38.27)	29.90 (25.10–35.50)	<0.001
Anion gap, mmol/L	14.00 (12.00–16.00)	13.00 (11.00–15.00)	14.00 (12.00–17.00)	<0.001
Bicarbonate, mmol/L	19.00 (16.00–23.00)	20.50 (17.00–23.00)	18.00 (15.00–23.00)	<0.001
Calcium, mmol/L	7.90 (7.30–8.50)	8.05 (7.40–8.60)	7.90 (7.30–8.40)	0.004
Chloridion, mmol/L	102.00 (97.00–105.25)	103.00 (99.00–106.00)	101.00 (97.00–105.00)	0.001
Sodium, mmol/L	137.00 (134.00–140.00)	137.00 (135.00–139.00)	137.00 (133.00–140.00)	0.347
Potassium, mmol/L	3.80 (3.40–4.10)	3.80 (3.40–4.10)	3.70 (3.40–4.20)	0.929
Creatinine, mg/dl	1.10 (0.80–1.80)	0.90 (0.70–1.30)	1.30 (0.80–2.10)	<0.001
BUN, mg/dl	21.00 (14.00–35.00)	19.00 (13.00–30.00)	24.00 (15.00–41.00)	<0.001
Glucose, mg/dl	117.00 (96.00–146.00)	116.00 (97.25–137.75)	118.00 (96.00–150.00)	0.487
ALT	48.00 (23.75–126.25)	44.00 (24.00–96.00)	51.00 (23.00–151.75)	0.031
Bilirubin	0.50 (0.30–1.00)	0.50 (0.40–0.90)	0.50 (0.30–1.00)	0.864
INR	1.20 (1.10–1.40)	1.10 (1.00–1.30)	1.30 (1.10–1.50)	<0.001
APTT	29.50 (26.10–35.00)	28.20 (25.30–32.88)	30.60 (26.70–36.50)	<0.001
**Scores**				
SOFA	9.00 (5.00–12.00)	7.00 (4.00–11.00)	10.00 (6.00–13.00)	<0.001
SAPSII	45.00 (35.00–57.00)	39.00 (30.00–50.00)	49.00 (40.00–61.00)	<0.001
Charlson index	6.00 (4.00–8.00)	5.00 (4.00–7.00)	7.00 (5.00–9.00)	<0.001
**Comorbidities, n (%)**				
Hypertension	522 (39.43%)	231 (46.02%)	291 (35.40%)	<0.001
Diabetes mellitus	470 (35.50%)	149 (29.68%)	321 (39.05%)	<0.001
Heart failure	513 (38.75%)	194 (38.65%)	319 (38.81%)	0.953
Atrial fibrillation	455 (34.37%)	242 (34.52%)	247 (36.81%)	0.376
AMI	398 (30.06%)	151 (30.08%)	247 (30.05%)	0.990
VHD	15 (1.13%)	8 (1.59%)	7 (0.85%)	0.216
Cardiomyopathy	85 (6.42%)	37 (7.37%)	48 (5.84%)	0.270
Pulmonary embolism	60 (4.53%)	23 (4.58%)	37 (4.50%)	0.946
Pulmonary hypertension	49 (3.70%)	14 (2.79%)	35 (4.26%)	0.170
COPD	357 (26.96%)	137 (27.29%)	220 (26.76%)	0.834
Renal diseases	385 (29.08%)	110 (21.91%)	275 (33.45%)	<0.001
Liver diseases	224 (16.92%)	69 (13.75%)	155 (18.86%)	0.016
Stroke	111 (8.38%)	27 (5.38%)	84 (10.22%)	0.002
Malignancy	163 (12.31%)	30 (5.98%)	133 (16.18%)	<0.001
**Therapies, n (%)**				
Mechanical ventilation	1,223 (92.37%)	450 (89.64%)	773 (94.04%)	0.003
RRT	85 (6.42%)	18 (3.59%)	67 (8.15%)	0.001
Vasopressor	828 (62.54%)	287 (57.17%)	541 (65.82%)	0.002
PCI	68 (5.14%)	33 (6.57%)	35 (4.26%)	0.064
**Therapies, n (%)**				
Assisted circulation	20 (1.51%)	9 (1.79%)	11 (1.34%)	0.511
ECMO	22 (1.66%)	8 (1.59%)	14 (1.70%)	0.880
Aspirin	596 (45.02%)	255 (50.80%)	341 (41.48%)	<0.001
Heparin	943 (71.22%)	384 (76.49%)	559 (68.00%)	<0.001
Transfusion of FFP	132 (9.97%)	38 (7.57%)	94 (11.44%)	0.023
Transfusion of platelet	85 (6.42%)	25 (4.98%)	60 (7.30%)	0.095

CCU, cardiac care unit; MICU, medical intensive care unit; HR, heart rate; RR, respiratory rate; MBP, mean blood pressure; WBC, white blood cell; BUN, blood urea nitrogen; ALT, alanine transaminase; INR, international normalized ratio; APTT, activated partial thromboplastin time; SOFA, the sequential organ failure assessment; SAPSII, the simplified acute physiology score II; AMI, acute myocardial infarction; VHD, valvular heart disease; COPD, chronic obstructive pulmonary disease; RRT, renal replacement therapy; PCI, percutaneous coronary intervention; ECMO, extracorporeal membrane oxygenation; FFP, fresh frozen plasma.

### 3.2. Associations between INR and all-cause mortality

**Figure 2:**
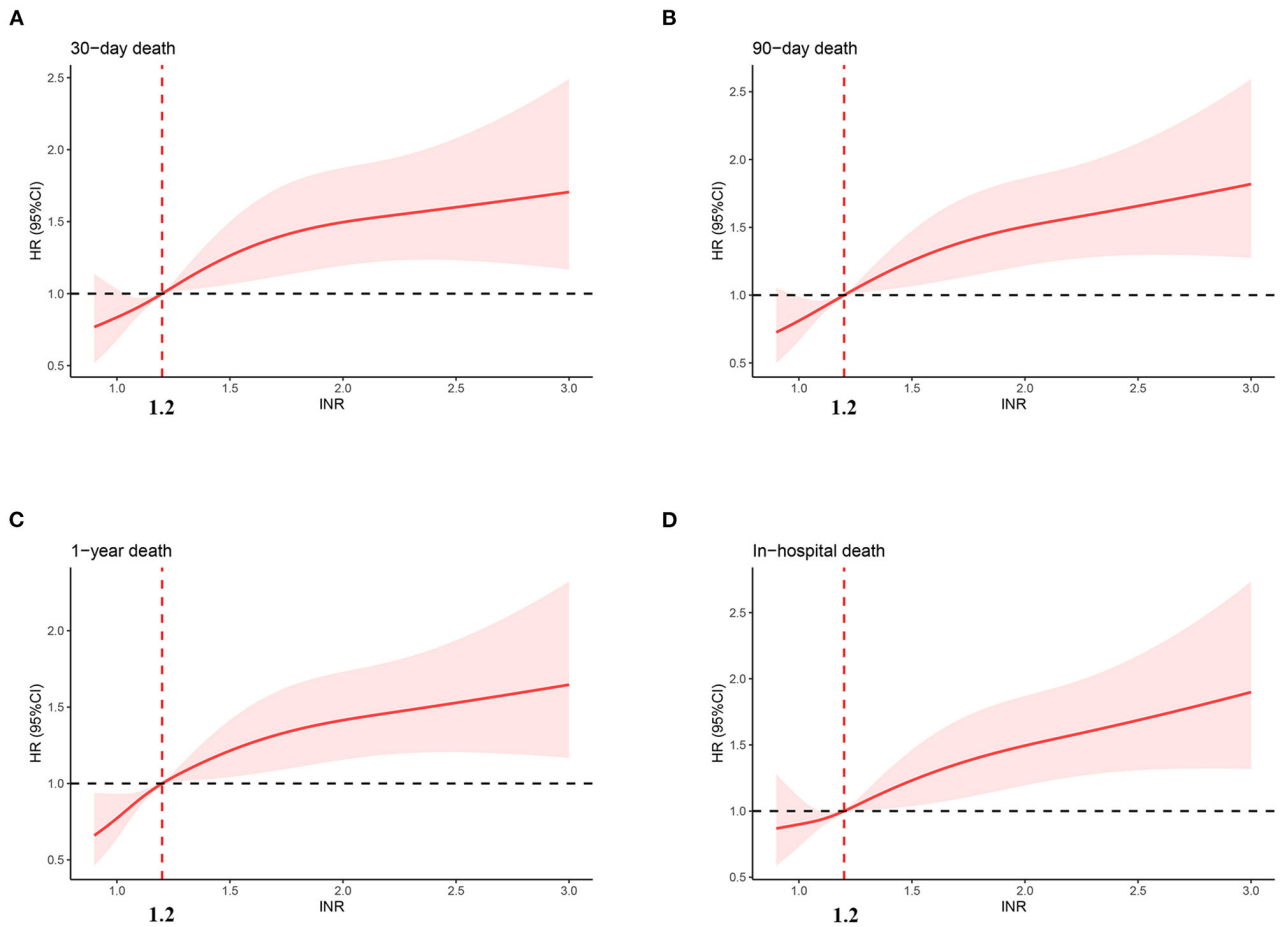
Association between INR and hazard ratio of 30-day **(A)**, 90-day **(B)**, 1-year **(C)**, and in-hospital **(D)** all-cause death. INR, international normalized ratio.

**Figure 3:**
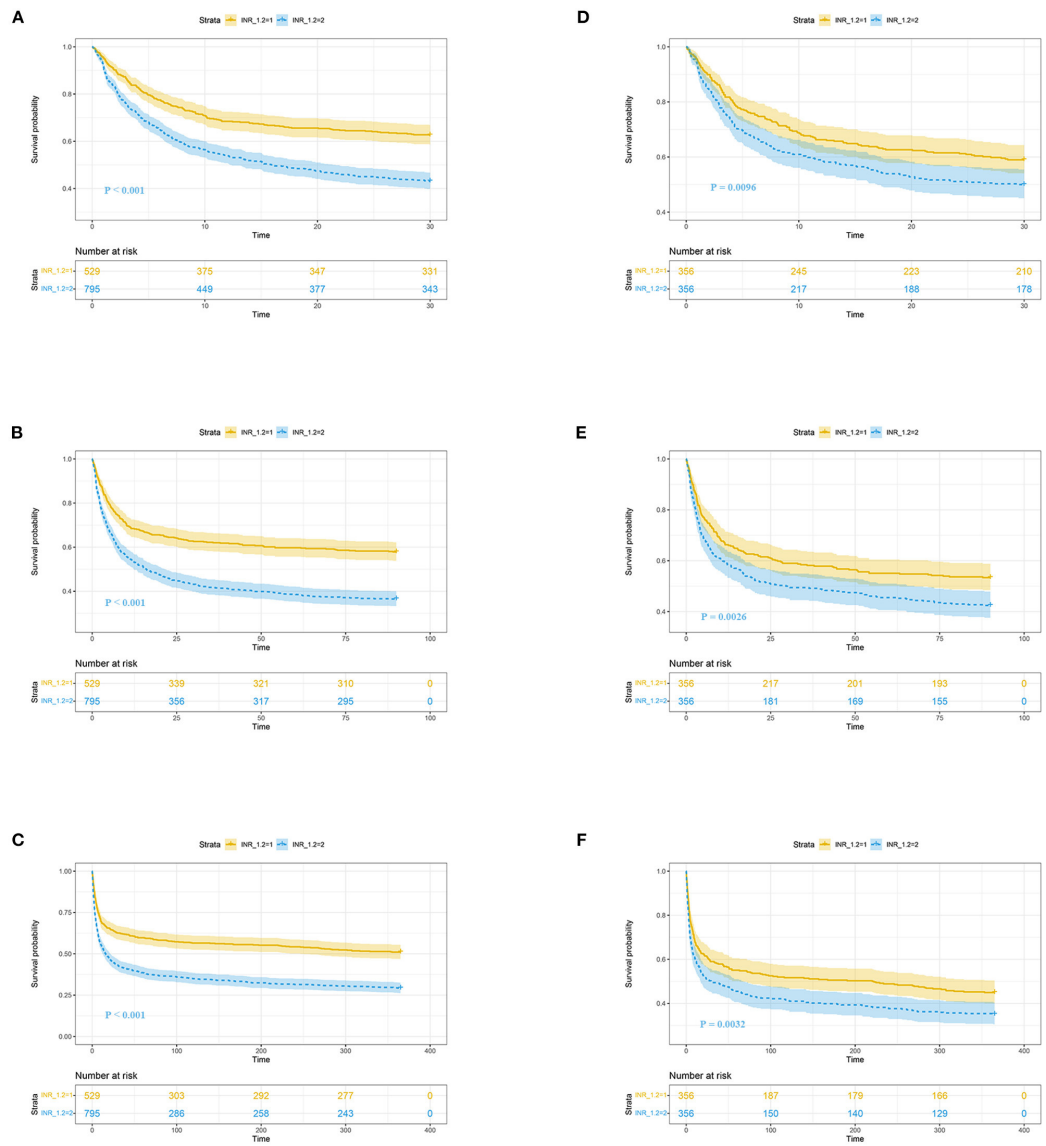
K-M survival curves of post-CA patients with high (blue, INR ≥ 1.2) and low (yellow, INR <1.2) INR at 30-day **(A, D)**, 90-day **(B, E)**, and 1-year **(C, F)** follow-up. **(A–F)** Reflect the results before and after propensity score matching, respectively. CA, cardiac arrest; INR, international normalized ratio.

**Figure 4:**
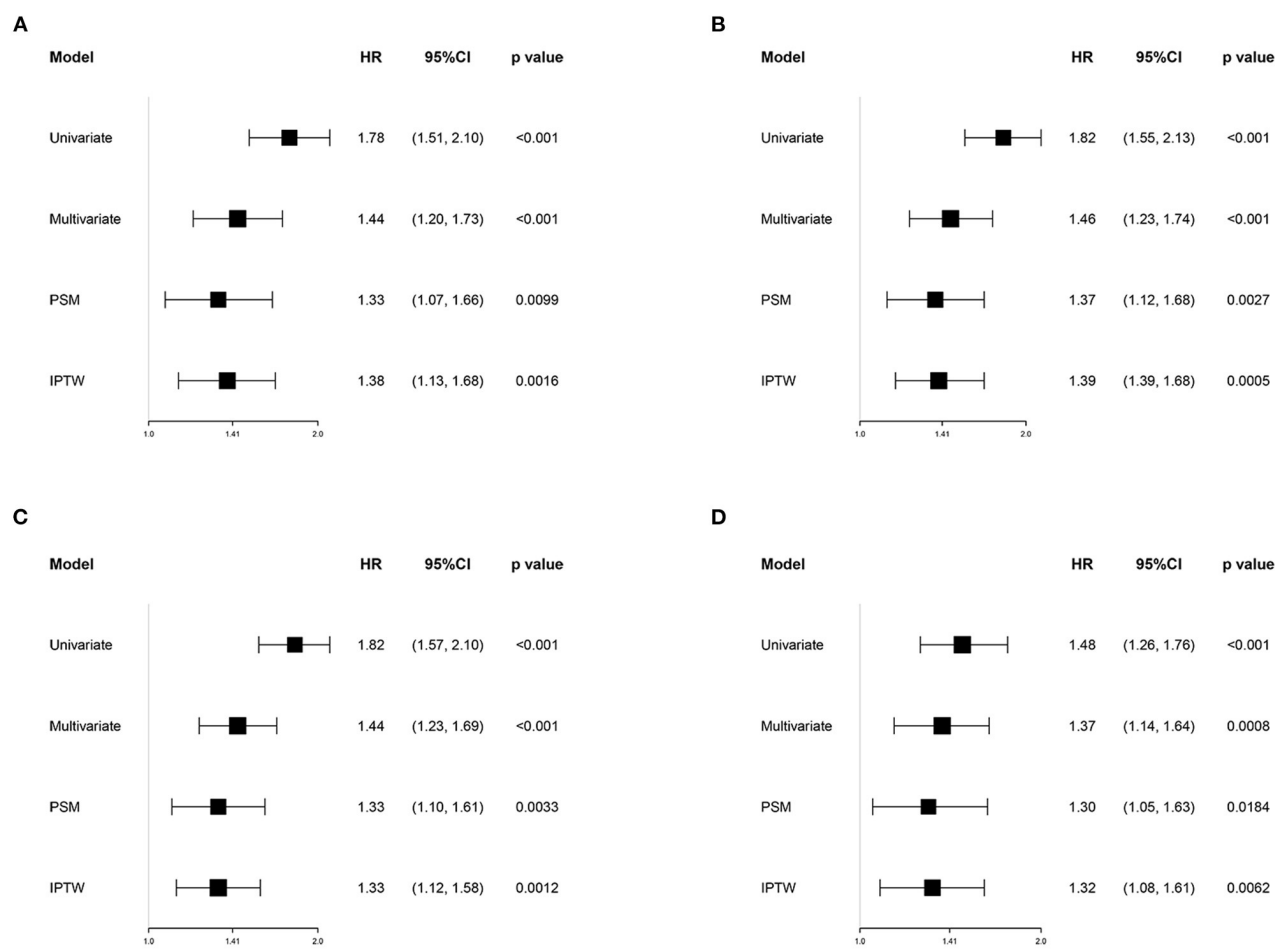
Association between INR group and 30-day **(A)**, 90-day **(B)**, 1-year **(C)**, and in-hospital **(D)** all-cause mortalities in different models. Univariate model was adjusted for none. Multivariate model was adjusted for age, gender, race, type of ICU for the first time, SOFA, SAPSII, Charlson comorbidity index, heart rate, mean blood pressure, anion gap, serum urea nitrogen, mechanical ventilation, vasopressors, renal replacement therapy, aspirin, warfarin, heparin, and the transfusion of fresh frozen plasma and platelets. HR, hazard ratio; CI, confidence interval; PSM, propensity score matching; IPTW, inverse probability of treatment weighting; SOFA, the sequential organ failure assessment; SAPSII, the simplified acute physiology score II.

Figure 2 shows the restricted cubic-spline model. A significant linear correlation existed between INR with all-cause mortality of post-CA patients, and the risk of death increased with elevated INR. Based on the result of restricted cubic-spline analysis, all subjects were divided into two groups according to the INR cutoff: low-INR group comprising 529 patients (INR <1.2), and high-INR group of 795 patients (INR ≥ 1.2). Consistently, K-M survival curves also illustrated that patients in the high-INR group were more likely to suffer from significantly elevated risks of all-cause death (*p* < 0.001; Figures 3A–C). To confirm whether the elevated INR was an independent risk factor for the increase in all-cause mortality of post-CA patients, we further conducted univariate and multivariate Cox regression analyses (Figure 4). In the univariate model, INR was strongly associated with a significant increase in 30-day (unadjusted HR = 1.78; 95% CI = 1.51–2.10; Figure 4A), 90-day (adjusted HR = 1.82; 95% CI = 1.55–2.13; Figure 4B), 1-year (unadjusted HR = 1.82; 95% CI = 1.57–2.10; Figure 4C), and in-hospital (unadjusted HR = 1.48; 95% CI = 1.26–1.76; Figure 4D) all-cause mortalities. After multivariate adjustment for the various confounders, the positive relationship between INR and 30-day (adjusted HR = 1.44; 95% CI = 1.20–1.73; Figure 4A), 90-day (adjusted HR = 1.46; 95% CI = 1.23–1.74; Figure 4B), 1-year (adjusted HR = 1.44; 95% CI = 1.23–1.69; Figure 4C), and in-hospital (adjusted HR = 1.37; 95% CI = 1.14–1.64; Figure 4D) all-cause mortalities remained significant.

### 3.3. Outcomes after PSM and IPTW

To reduce the influence of confounding bias, the PSM and IPTW analyses were also performed in our studies. After PSM, 356 high-INR and 356 low-INR patients were enrolled in the final analysis. Almost all covariates were evenly distributed across the two groups, except for potassium and the transfusion of FFP (Table 2; Supplementary Figure 1). In the PSM cohort, the survival probability in the hospital and 30-day, 90-day, and 1-year after discharge of patients with post-CA was significantly higher in the low-INR than high-INR group (Figures 3D–F). The elevated INR still contributed to increased 30-day (HR = 1.33; 95% CI = 1.07–1.66), 90-day (HR = 1.37; 95% CI = 1.12–1.66), 1-year (HR = 1.33; 95% CI = 1.10–1.61), and in-hospital (HR = 1.30; 95% CI = 1.05–1.63) all-cause mortalities in the PSM analysis, as shown in Figure 4. After IPTW, no significant difference existed in baseline levels between the high-INR and low-INR groups (Supplementary Figure 1). The association of high INR level with excess all-cause mortality remained significant (Figure 4).

**Table 2:** The baseline characteristics of post-CA patients grouped by INR before and after PSM.

**Variables**	**Before PSM**			**After PSM**		

	**INR**<**1.2**	**INR** ≥**1.2**	**SMD**	**INR**<**1.2**	**INR** ≥**1.2**	**SMD**
N	529	795		356	356	
Age, years	65.45 (54.34–76.23)	69.16 (56.94–80.38)	0.192	67.56 (58.36–78.77)	69.38 (55.72–80.37)	0.013
Male, %	309 (58.41%)	512 (64.40%)	0.123	210 (58.99%)	197 (55.34%)	0.074
**Race, %**			0.220			0.083
White	291 (55.01%)	468 (58.87%)		201 (56.46%)	191 (53.65%)	
Black	41 (7.75%)	98 (12.33%)		32 (8.99%)	40 (11.24%)	
Asian	14 (2.65%)	22 (2.77%)		12 (3.37%)	11 (3.09%)	
Other	183 (34.59%)	207 (26.04%)		111 (31.18%)	114 (32.02%)	
**First care unit**			0.169			0.011
CCU	231 (43.67%)	301 (37.86%)		153 (42.98%)	151 (42.42%)	
MICU	164 (31.00%)	310 (38.99%)		112 (31.46%)	113 (31.74%)	
Other	134 (25.33%)	184 (23.14%)		91 (25.56%)	92 (25.84%)	
**Vital signs**						
HR, beats/minute	78.52 (66.79–91.69)	84.22 (73.84–98.21)	0.371	80.61 (69.16–92.94)	79.83 (70.86–91.05)	0.002
RR, times/minute	19.60 (17.30–22.77)	20.12 (17.55–23.25)	0.102	19.66 (17.39–23.19)	19.60 (17.27–22.59)	0.032
MBP, mmHg	78.84 (73.28–87.00)	75.91 (69.33–82.71)	0.355	77.98 (72.20–85.29)	79.11 (72.49–85.50)	0.039
Temperature, °C	36.74 (36.33–37.08)	36.71 (36.24–37.11)	0.054	36.77 (36.39–37.09)	36.73 (36.32–37.15)	0.008
SpO2, %	98.00 (96.39–99.15)	97.67 (95.98–98.99)	0.189	97.82 (96.22–99.01)	98.00 (96.37–99.15)	0.027
Weight, kg	80.00 (67.90–94.30)	80.00 (66.60–96.15)	0.016	79.00 (66.20–94.00)	79.00 (64.47–95.53)	0.047
Urine amount, L	1.50 (0.90–2.37)	1.14 (0.52–1.90)	0.232	1.33 (0.81–2.18)	1.29 (0.81–2.06)	0.020
**Laboratory tests**						
Hemoglobin, g/dL	11.20 (9.10–13.00)	9.40 (8.00–11.20)	0.558	10.40 (8.60–12.40)	10.35 (8.80–12.00)	0.021
Platelet, K/μl	171.00 (134.00–225.00)	157.00 (108.00–223.00)	0.116	171.00 (126.00–231.25)	167.50 (124.00–223.00)	0.023
WBC, K/μl	9.80 (7.00–13.10)	10.10 (7.10–14.00)	0.133	10.05 (6.90–13.62)	9.95 (7.20–13.20)	<0.001
Hematocrit	33.80 (27.80–38.70)	29.20 (24.90–34.30)	0.496	31.55 (26.00–37.02)	31.35 (27.02–36.23)	0.018
**Laboratory tests**						
Anion gap, mmol/L	13.00 (11.00–15.00)	14.00 (12.00–17.00)	0.324	13.00 (11.00–16.00)	13.00 (11.00–16.00)	0.003
Bicarbonate, mmol/L	20.00 (16.00–23.00)	19.00 (15.00–23.00)	0.164	19.00 (16.00–23.00)	19.00 (16.00–23.00)	0.020
Calcium, mmol/L	8.00 (7.40–8.50)	7.90 (7.30–8.40)	0.120	8.00 (7.40–8.50)	8.00 (7.40–8.50)	0.015
Chloridion, mmol/L	102.00 (98.00–105.00)	102.00 (97.00–106.00)	0.121	102.00 (97.75–106.00)	102.00 (98.00–106.00)	0.011
Sodium, mmol/L	137.00 (135.00–140.00)	137.00 (133.00–140.00)	0.096	137.00 (134.00–139.00)	137.00 (134.00–140.00)	0.048
Potassium, mmol/L	3.70 (3.30–4.10)	3.80 (3.40–4.20)	0.238	3.80 (3.40–4.12)	3.70 (3.40–4.00)	0.103
Creatinine, mg/dl	0.90 (0.70–1.40)	1.30 (0.85–2.10)	0.325	1.00 (0.70–1.60)	1.05 (0.70–1.52)	0.005
BUN, mg/dl	17.00 (12.00–26.00)	24.00 (15.00–41.00)	0.487	19.00 (14.00–29.00)	20.00 (14.00–30.00)	0.011
Glucose, mg/dl	119.00 (101.00–144.00)	116.00 (95.00–147.00)	0.001	121.00 (101.75–148.00)	116.00 (96.00–145.00)	0.011
ALT	57.00 (27.00–138.00)	42.00 (22.00–119.00)	0.179	51.00 (24.00–137.00)	42.00 (22.00–95.50)	0.026
Bilirubin	0.40 (0.30–0.70)	0.60 (0.40–1.20)	0.350	0.50 (0.30–0.80)	0.50 (0.30–0.80)	0.043
APTT	27.30 (24.52–30.20)	31.90 (27.70–38.10)	0.545	28.20 (25.50–31.50)	29.25 (25.90–33.50)	0.040
Charlson index	5.00 (3.00–7.00)	7.00 (5.00–9.00)	0.468	6.00 (4.00–8.00)	6.00 (4.00–8.00)	0.017
**Comorbidities, n (%)**						
Hypertension	247 (46.69%)	275 (34.59%)	0.248	157 (44.10%)	159 (44.66%)	0.011
Diabetes mellitus	165 (31.19%)	305 (38.36%)	0.151	130 (36.52%)	126 (35.39%)	0.023
Heart failure	175 (33.08%)	338 (42.52%)	0.195	130 (36.52%)	141 (39.61%)	0.064
Atrial fibrillation	134 (25.33%)	321 (40.38%)	0.325	116 (32.58%)	119 (33.43%)	0.018
AMI	168 (31.76%)	230 (28.93%)	0.062	110 (30.90%)	105 (29.49%)	0.031
VHD	5 (0.95%)	10 (1.26%)	0.030	5 (1.40%)	6 (1.69%)	0.023
Cardiomyopathy	28 (5.29%)	57 (7.17%)	0.078	19 (5.34%)	19 (5.34%)	<0.001
Pulmonary embolism	16 (3.02%)	44 (5.53%)	0.124	13 (3.65%)	12 (3.37%)	0.015
Pulmonary hypertension	8 (1.51%)	41 (5.16%)	0.204	8 (2.25%)	13 (3.65%)	0.083
COPD	141 (26.65%)	216 (27.17%)	0.012	112 (31.46%)	104 (29.21%)	0.049
Renal diseases	104 (19.66%)	281 (35.35%)	0.357	93 (26.12%)	85 (23.88%)	0.052
**Comorbidities, n (%)**						
Liver diseases	42 (7.94%)	182 (22.89%)	0.423	36 (10.11%)	30 (8.43%)	0.058
Stroke	45 (8.51%)	66 (8.30%)	0.007	34 (9.55%)	30 (8.43%)	0.039
Malignancy	43 (8.13%)	120 (15.09%)	0.219	37 (10.39%)	35 (9.83%)	0.019
**Therapies, n (%)**						
Mechanical ventilation	479 (90.55%)	744 (93.58%)	0.113	330 (92.70%)	336 (94.38%)	0.069
RRT	24 (4.54%)	61 (7.67%)	0.131	20 (5.62%)	20 (5.62%)	<0.001
Vasopressor	292 (55.20%)	536 (67.42%)	0.253	223 (62.64%)	224 (62.92%)	0.006
PCI	40 (7.56%)	28 (3.52%)	0.177	18 (5.06%)	20 (5.62%)	0.025
Assisted circulation	8 (1.51%)	12 (1.51%)	<0.001	8 (2.25%)	5 (1.40%)	0.063
ECMO	5 (0.95%)	17 (2.14%)	0.097	5 (1.40%)	5 (1.40%)	<0.001
Aspirin	246 (46.50%)	350 (44.03%)	0.050	170 (47.75%)	165 (46.35%)	0.028
Heparin	413 (78.07%)	530 (66.67%)	0.257	260 (73.03%)	257 (72.19%)	0.019
**Therapies, n (%)**						
Transfusion of FFP	21 (3.97%)	111 (13.96%)	0.355	21 (5.90%)	31 (8.71%)	0.108
Transfusion of platelet	17 (3.21%)	68 (8.55%)	0.228	16 (4.49%)	23 (6.46%)	0.086
**Scores**						
SOFA	8.00 (4.00–11.00)	10.00 (6.00–13.00)	0.448	9.00 (5.00–12.00)	8.00 (5.00–12.00)	0.003
SAPSII	41.00 (31.00–52.00)	48.00 (38.00–60.00)	0.461	45.50 (35.00–55.00)	43.00 (36.00–55.25)	0.010

PSM, propensity score matching; CCU, cardiac care unit; MICU, medical intensive care unit; HR, heart rate; RR, respiratory rate; MBP, mean blood pressure; WBC, white blood cell; BUN, blood urea nitrogen; ALT, alanine transaminase; INR, international normalized ratio; APTT, activated partial thromboplastin time; SOFA, the sequential organ failure assessment; SAPSII, the simplified acute physiology score II; AMI, acute myocardial infarction; VHD, valvular heart disease; COPD, chronic obstructive pulmonary disease; RRT, renal replacement therapy; PCI, percutaneous coronary intervention; ECMO, extracorporeal membrane oxygenation; FFP, fresh frozen plasma.

### 3.4. Subgroup analyses

Subgroup analysis of the association between INR and 1-year all-cause mortality was completed on the training cohort by using demographics, severity of illness scores, and several comorbidities, and results are presented in Table 3. Overall, the positive correlation between INR and all-cause mortality was generally consistent across subgroups, with higher INR associated with higher mortality. No significant interaction was observed in most strata (*p* = 0.0679–0.9719), except for AMI (*p* = 0.0434). Among post-CA patients, patients complicated with AMI tended to have higher risks of 1-year all-cause death for high INR than that of patients without AMI.

**Table 3:** Subgroup analysis of the association between INR and 1-year all-cause mortality.

**Variables**	**N**	**INR**		***p* for interaction**

		<**1.2**	≥**1.2**	
**Gender**				0.4065
Female	503	1 (ref)	1.70 (1.36, 2.13)	
Male	821	1 (ref)	1.94 (1.59, 2.36)	
**Age**				0.9790
<65	589	1 (ref)	1.75 (1.39, 2.20)	
≥ 65	735	1 (ref)	1.81 (1.49, 2.19)	
**Race**				0.5606
White	759	1 (ref)	1.92 (1.56, 2.36)	
Black	139	1 (ref)	2.59 (1.54, 4.35)	
Asian	36	1 (ref)	1.47 (0.63, 3.45)	
Other	390	1 (ref)	1.65 (1.28, 2.11)	
**Hypertension**				0.2826
No	802	1 (ref)	1.94 (1.60, 2.35)	
Yes	522	1 (ref)	1.58 (1.25, 2.00)	
**Diabetes**				0.2281
No	854	1 (ref)	1.65 (1.37, 1.99)	
Yes	470	1 (ref)	2.13 (1.66, 2.74)	
**AMI**				0.0434
No	926	1 (ref)	1.63 (1.37, 1.94)	
Yes	398	1 (ref)	2.33 (1.78, 3.06)	
**Atrial fibrillation**				0.4783
No	869	1 (ref)	1.85 (1.54, 2.21)	
Yes	455	1 (ref)	1.73 (1.33, 2.26)	
**VHD**				0.1978
No	1,309	1 (ref)	1.83 (1.58, 2.13)	
Yes	15	1 (ref)	1.01 (0.38, 5.41)	
**Pulmonary hypertension**				0.0679
No	1,275	1 (ref)	1.77 (1.52, 2.06)	
Yes	49	1 (ref)	5.67 (1.35, 23.73)	
**Pulmonary embolism**				0.0769
No	1,264	1 (ref)	1.87 (1.61, 2.17)	
Yes	60	1 (ref)	1.03 (0.66, 1.88)	
**Heart failure**				0.1236
No	811	1 (ref)	1.65 (1.38, 1.98)	
Yes	513	1 (ref)	2.28 (1.76, 2.95)	
**COPD**				0.5496
No	967	1 (ref)	1.76 (1.48, 2.09)	
Yes	357	1 (ref)	1.97 (1.48, 2.63)	
**Renal diseases**				0.8506
No	939	1 (ref)	1.73 (1.45, 2.06)	
Yes	385	1 (ref)	1.94 (1.45, 2.60)	
**Liver diseases**				0.1442
No	1,100	1 (ref)	1.72 (1.47, 2.02)	
Yes	224	1 (ref)	2.56 (1.56, 4.19)	
**Cardiomyopathy**				0.5052
No	1,239	1 (ref)	1.80 (1.55, 2.09)	
Yes	85	1 (ref)	2.35 (1.17, 4.72)	
**Stroke**				0.1069
No	1,213	1 (ref)	1.88 (1.60, 2.19)	
Yes	111	1 (ref)	1.30 (0.83, 2.02)	
**Malignancy**				0.7688
No	1,161	1 (ref)	1.75 (1.49, 2.05)	
Yes	163	1 (ref)	2.01 (1.33, 3.04)	
**SOFA**				0.3772
<5	262	1 (ref)	1.47 (1.01, 2.14)	
≥5	1,062	1 (ref)	1.76 (1.50, 2.07)	
**SAPSII**				0.9719
<40	469	1 (ref)	1.63 (1.24, 2.15)	
≥40	855	1 (ref)	1.64 (1.38, 1.96)	

INR, international normalized ratio; AMI, acute myocardial infarction; VHD, valvular heart disease; COPD, chronic obstructive pulmonary disease; SOFA, the sequential organ failure assessment; SAPSII, the simplified acute physiology score II.

### 3.5. Sensitivity analyses and validation

Certain differences in clinical management existed across cardiogenic or non-cardiogenic post-CA patients, which may alter coagulation status and affect INR modification. Accordingly, we conducted sensitivity analyses to include only post-CA patients with cardiac diseases or ones without cardiac diseases, respectively. Similar to main analyses, INR was still a significant and robust predictor for 1-year all-cause mortality of post-CA patients with or without cardiac diseases (Table 4).

**Table 4:** Sensitivity analysis of the association between INR and 1-year all-cause mortality in post-CA patients with or without cardiac diseases.

	***N***	**Crude**		**Model I**		**Model II**	

		**HR (95% CI)**	***p*** **value**	**HR (95% CI)**	***p*** **value**	**HR (95% CI)**	***p*** **value**
**Without cardiac diseases**							
INR group	627						
<1.2	257	1 (ref)		1(ref)		1(ref)	
≥1.2	370	1.50 (1.22, 1.84)	<0.001	1.57 (1.28, 1.93)	<0.001	1.27 (1.01, 1.60)	0.0395
**With cardiac diseases**							
INR group	697						
<1.2	272	1 (ref)		1 (ref)		1 (ref)	
≥1.2	425	2.23 (1.80, 2.77)	<0.001	2.14 (1.72, 2.66)	<0.001	1.63 (1.29, 2.06)	<0.001

Crude model was adjusted for none. Model I was adjusted for age, gender, and race. Model II was adjusted for age, gender, race, type of intensive care unit for the first time, SOFA, SAPSII, Charlson comorbidity index, heart rate, mean arterial pressure, anion gap, blood urea nitrogen, mechanical ventilation, vasopressors, renal replacement therapy, aspirin, heparin, and the transfusion of fresh frozen plasma and platelets.

CA, cardiac arrest; HR, hazard ratio; INR, international normalized ratio; SOFA, the sequential organ failure assessment; SAPSII, the simplified acute physiology score II.

We then performed an external validation in the validation cohort from the eICU database, and the baseline characteristics of subjects are presented in Supplementary Table 1. In the crude model without adjusting for covariates, high INR was related to the elevated in-hospital mortality (unadjusted OR = 2.07; 95% CI = 1.63–2.64; Table 5). In model I after adjusting for age, gender, and race, high INR was also associated with increased in-hospital mortality (adjusted OR = 2.03; 95% CI = 1.59–2.60; Table 5). Model II further adjusted for other confounders, including the type of ICU for the first time, APACHE-IV scores, heart rate, MBP, anion gap, BUN, the use of mechanical ventilation and RRT, the admission of vasopressors, aspirin, and heparin, and the transfusion of FFP and platelets. Elevated INR still can independently predict the high in-hospital mortality (adjusted OR = 1.34; 95% CI = 1.01–1.78; Table 5).

**Table 5:** The logistics regression analyses of INR for predicting 1-year all-cause mortality of post-CA patients in validation cohort.

	**Crude**		**Model I**		**Model II**	

	**OR (95% CI)**	***p*** **value**	**OR (95% CI)**	***p*** **value**	**OR (95% CI)**	***p*** **value**
INR	1.39 (1.25, 1.56)	<0.001	1.38 (1.23, 1.54)	<0.001	1.19 (1.07, 1.32)	0.0013
**INR group**						
<1.2	1 (ref)		1(ref)		1(ref)	
≥ 0.2	2.07 (1.63, 2.64)	<0.001	2.03 (1.59, 2.60)	<0.001	1.34 (1.01, 1.78)	0.0400

Crude model was adjusted for none. Model I was adjusted for age, gender, and race. Model II was adjusted for age, gender, race, type of intensive care unit for the first time, APACHE-IV scores, heart rate, mean arterial pressure, anion gap, blood urea nitrogen, the use of mechanical ventilation and renal replacement therapy, the admission of vasopressors, aspirin, and heparin, and the transfusion of fresh frozen plasma and platelets.

CA, cardiac arrest; OR, odds ratio; INR, international normalized ratio; APACHE-IV, acute physiology and chronic health evaluation IV.

### 3.6. Predictive values of INR and some severity scores for 1-year all-cause mortality

**Figure 5:**
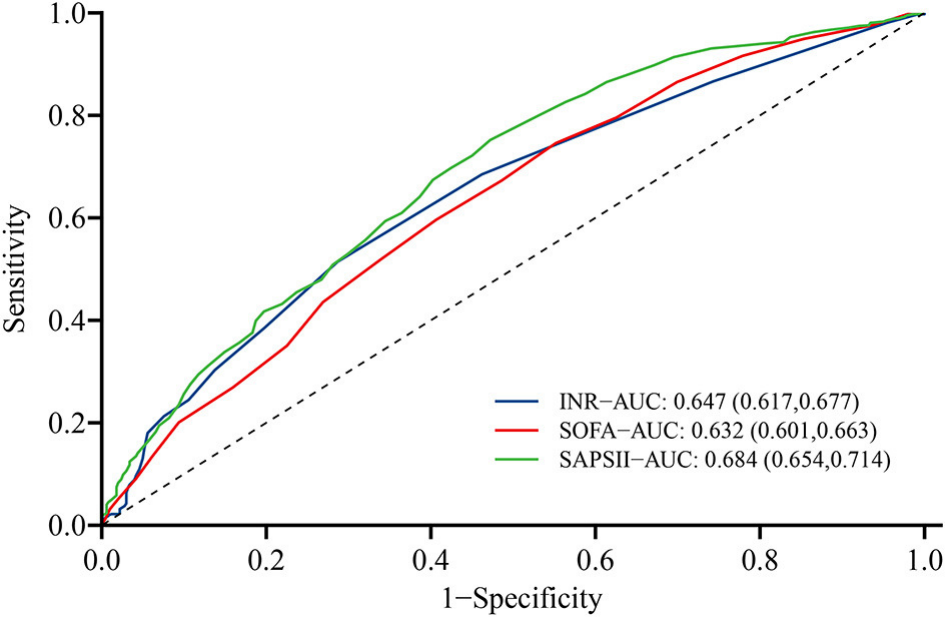
Receiver operating characteristic curve of INR (blue), SOFA score (green), and SAPSII score (red) for predicting 1-year all-cause mortality of post-CA patients. CA, cardiac arrest; SOFA, the sequential organ failure assessment; SAPSII, the simplified acute physiology score II.

ROC curves were obtained to evaluate the predictive performance of INR and some severity scoring systems (SOFA and SAPSII) for 1-year all-cause mortality, as shown in Figure 5. Compared with SAPSII score (area under ROC = 0.684; 95% CI: 0.654–0.714), the INR (area under ROC = 0.647, 95% CI: 0.617–0.677) had the slightly worse power for predicting the 1-year all-cause mortality of patients with post-CA, whereas the performance was comparable with SOFA score (area under ROC = 0.632; 95% CI = 0.601–0.663). Besides, compared with SOFA and SASPII scores, INR displayed a relatively higher specificity (71.3%) but lower sensitivity (51.5%) for predicting 1-year all-cause mortality in post-CA patients with 1.2 as the cutoff point (Supplementary Table 2).

## 4. Discussion

The clinical data of MIMIC-IV and eICU databases were analyzed retrospectively to evaluate the possibility of INR predicting the prognosis of post-CA patients. Results showed that the INR within 24 h after admission to ICU was an independent risk factor for 30-day, 90-day, 1-year, and in-hospital all-cause mortalities of post-CA patients. First, we explored the potential relationship between INR and all-cause mortality by restricted cubic-spline analysis. A significant linear positive correlation was found between INR and all-cause mortality, and the risk of all-cause death increased significantly when INR was <1.2 (HR > 1). K-M survival curve and Cox regression analysis further confirmed that the all-cause mortality in patients with high INR was significantly higher than that in patients with low INR, which was still robust in the subgroup analyses, PSM and IPTW analyses, and validation cohort. Overall, this study illustrated that INR was helpful for the risk stratification of post-CA patients to identify high-risk ones and contribute to the clinical management of patients.

Several studies have demonstrated that a high level of INR is strongly associated with poor prognosis in various diseases. Zheng et al. (22) illustrated that INR is positively correlated with all-cause mortality in patients with sepsis, with an adjusted odds ratio (OR) of 1.86 (95% CI: 1.37–2.52) for in-hospital mortality, and an adjusted HR of 1.47 (95% CI: 1.24–1.74) for 1-year mortality. Among patients with coronary artery disease, an increased risk of all-cause mortality has been found in those with high levels of INR (INR > 1.06) during a median follow-up of 5.25 years (23). Ki-Hong et al. (24) also reported that prothrombin time–INR prolongation was associated with poor in-hospital survival (adjusted OR = 0.28; 95% CI = 0.11–0.69) in adult out-of-hospital CA with cardiac etiology. In this multicenter cross-sectional study, only the relation between INR and survival at discharge is analyzed. The survival time of subjects, which is significant for evaluating the condition and severity of patients, is not considered. The association between INR and long-term prognosis of post-CA patients is also not explored due to the lack of follow-up after discharge. In the present study, we systematically and comprehensively analyzed the relationship of high level of INR with the short-term (30-day, 90-day, and in-hospital mortalities) and long-term (1-year mortality) outcome of post-CA patients, considering the occurrence of outcome events and the occurrence time of events. The baseline data we collected were also more comprehensive and complete, including demographic data, vital signs, laboratory tests, disease-severity scores, treatment measures, etc. They were adjusted by multivariate Cox regression analysis, PSM and IPTW analyses, and other statistical methods, which were conducive to enhancing the reliability of our results.

As a prognostic biomarker, INR may have the following advantages. First, INR is calculated according to the prothrombin time and the international sensitivity index of the assay reagent. Compared with prothrombin time, INR presents better consistency across different medical institutions, and the measurement results are comparable (25). Second, INR can be detected readily and quickly in most hospitals, with the strengths of timeliness and low cost (26). Last, coagulation disorders in CA patients are being commonly recognized, especially abnormalities in exogenous coagulation pathways triggered by tissue factors (27). As a commonly used laboratory indicator reflecting changes in exogenous coagulation pathways, a certain theoretical basis exists for INR to predict the prognosis of post-CA patients.

The pathophysiological mechanism by which elevated INR is associated with poor prognosis in post-CA patients is unclear, but multiple possibilities could explain the increased INR in post-CA patients. First, the consumption of coagulation factors can lead to increased INR after the excessive activation of the coagulation system. Direct damages to cells and tissues during the no blood flow and perfusion period after CA, ischemia–reperfusion injury after spontaneous circulation recovery, and overactive neurohormones can all induce endothelial-cell dysfunction and trigger the expression of tissue factors. These phenomena can generate thrombin and cause coagulation by a series of cascade reaction (28). Moreover, some well-known platelet activators such as hypoxia, ischemia, thrombin, catecholamines, etc. can be induced after CA, thereby contributing to platelet hyperactivation and leading to a thrombo-inflammatory state characterized by the expression of tissue factors and thrombin produce (9). Tissue factor-mediated coagulation activation and platelet activation are also the main triggers of DIC, which are characterized by systemic-coagulation activation and insufficient endogenous fibrinolysis, leading to intravascular fibrin formation and microvascular thrombosis (29). The prevalence of overt DIC in post-CA patients is not low, about 42–54%, and a higher DIC score is closely related to increase in-hospital mortality in patients with out-of-hospital CA (OR = 1.89; 95% CI = 1.48–2.41) (30). Prolonged prothrombin time and increased INR are fairly common in liver dysfunction, and another possible explanation for increased INR is hypoxic liver injury after CA. Due to the loss of blood supply, multiple tissues and organs including the liver suffer from severe ischemia and hypoxia after CA, resulting in centrilobular liver cell necrosis and acute liver injury. Furthermore, reperfusion injury after the recovery of spontaneous circulation due to timely cardiopulmonary resuscitation is an important cause of liver injury (31). According to Roedl et al. (32), the prevalence of hypoxic liver injury in out-of-hospital and in-hospital CA patients have similar values of 21 and 19%, respectively. The occurrence of hypoxic liver injury can predict the possible terrible outcome of post-CA patients. Compared with those without hypoxic liver injury, the 1-year mortality of post-CA patients complicated with hypoxic liver injury is significantly higher (61 vs. 49%, *p* < 0.001).

Herein, subgroup analysis was conducted for sensitivity analysis to improve the robustness of our research results. In all subgroups stratified by age, gender, race, comorbidities, and disease-severity scores, the association of high-level INR with increased 1-year all-cause mortality of post-CA patients was a consistent finding. The conclusion that INR was positively associated with mortality still stood in post-CA patients accompanied with stroke, pulmonary embolism, or VHD, but it was not statistically significant, which may be attributed to the limited sample size after stratification. We also found that INR interacted with AMI on the 1-year all-cause death in post-CA patients (p for interaction <0.05). In post-CA patients with AM, the relationship between INR ≥ 1.2 and the increase in all-cause mortality was more significant. One possible explanation was that AMI can promote inflammatory response and neurohormone activation, which were previously considered as important triggers leading to the activation of coagulation system, consumption of coagulation factors, and increase in INR (17).

A series of disease severity scoring systems, such as SOFA and SAPSII scores, have been developed and applied satisfactorily to assess the severity of critically ill patients and predict their prognosis, including the outcome of post-CA patients. Matsuda et al. (33) found that patients with post-CA syndrome who survive or have favorable neurological outcome tend to have a lower SOFA score, and SOFA score at admission is an independent predictor of 30-day survival (OR = 0.68; 95% CI = 0.59–0.78). To evaluate the predictive efficiency of INR for the prognosis of post-CA patients, the present study also performed ROC analysis and calculated the area under the curve to compare the performance of INR with SOFA and SAPSII scores in predicting the 1-year all-cause mortality. Overall, the performance of INR was roughly equal to SOFA score but slightly inferior to SAPSII score. However, in clinical practice, acquiring complete and systemic SOFA or SAPSII scores in a timely manner is often difficult. SOFA score (34) becomes available only after the collection of oxygenation index, platelet count, serum bilirubin concentration, serum creatinine concentration, urine volume, Glasgow score, and cardiovascular function. SAPSII score (35) also requires the data of vital signs (e.g., heart rate and systolic blood pressure) and of laboratory tests (e.g., serum sodium and potassium), thereby limiting the clinical convenience and timeliness of these scores to some extent. By contrast, INR is a routine indicator used in most critically ill patients. INR is characterized by being easy and quick to obtain and may have great clinical practicality. We must also note that the discrimination for 1-year all-cause mortality with INR, SOFA and SAPSII scores was not so satisfactory, with the relatively low area under the curve of ROC (0.632–0.684). This is consistent with the previous study. Choi et al. (36) showed that the area under the curve for SOFA and SAPSII scores to predict 30-day mortality of post-CA patients were 0.641 (0.564–0.712) and 0.686 (0.612–0.755), respectively. Besides, for predicting the 1-year all-cause mortality, INR presented the higher specificity, but SOFA and SAPSII scores tended to be higher sensitivity. This showed that in clinical practice, SOFA and SAPSII scores can identify more high-risk patients from critically ill patients, while the application of INR will help reduce the possibility that some low-risk patients may be misjudged as high-risk. And the combination of INR and SOFA or SAPSII score may well be an option to be considered.

The present study had also some limitations. First, this research was a retrospective cohort study, so selection bias was inevitable, and it was difficult to ensure that all variables were evenly distributed across groups. Although multiple regression analysis, PSM, and IPTW were conducted to adjust the confounder and thus improve the reliability of our findings, a prospective cohort study was meaningful to perform for evaluating the relationship between INR and the prognosis of post-CA patients. Second, the relevant data of this study were extracted from a public database, and some recording errors were possible. Third, the research subjects we included were patients admitted to the ICU in the BIDMC from 2008 to 2019. With such a great time span, the diagnosis and treatment may be updated in this period, which may affect the prognosis. Last, the underlying mechanism between elevated INR and increased mortality in post-CA patients remains unclear, and further research is necessary.

## 5. Conclusions

High levels of INR were closely associated with the poor short- and long-term prognosis in post-CA patients, including 30-day, 90-day, 1-year, and in-hospital all-cause mortalities. INR is expected to be a simple and effective prognostic evaluation indicator.

## Data availability statement

Publicly available datasets were analyzed in this study. The data can be extracted from MIMIC-IV database (https://physionet.org/content/mimiciv/2.0/) after passing on the required courses and obtaining the authorization.

## Ethics statement

Ethical review and approval was not required for the study on human participants in accordance with the local legislation and institutional requirements. Written informed consent for participation was not required for this study in accordance with the national legislation and the institutional requirements.

## Author contributions

LZ and QC conceived and designed the study. YT extracted and analyzed the clinical data. JS drafted the manuscript and assisted in the statistical analysis. ZY, BL, and BP participated in the implementation of statistical methods and put forward constructive suggestions. JM, XZ, and YF reviewed the study and participated in the interpretation of the results. All authors approved the final manuscript.
