# Supplementary material for: Association between prothrombin time-international normalized ratio and prognosis of post-cardiac arrest patients: A retrospective cohort study
Source: Front Public Health. 2023 Jan 20;11:1112623. doi: 10.3389/fpubh.2023.1112623 (PMC9895096; doi:10.3389/fpubh.2023.1112623)
Supplement: Supplementary file 1 [file Table_1.DOCX]

Supplementary Material

Association between Prothrombin Time-International Normalized Ratio and Prognosis of Post‑cardiac Arrest Patients: A retrospective cohort study

Yiyang Tang^#^, Jing Sun^#^, Zaixin Yu, Benhui Liang, Baohua Peng, Jing Ma, Xiaofang Zeng, Yilu Feng, Qin Chen^*^, Lihuang Zha^*^

*** Correspondence:**

Qin Chen, chenqin990821@163.com

Lihuang Zha, zhalihuang@csu.edu.cn


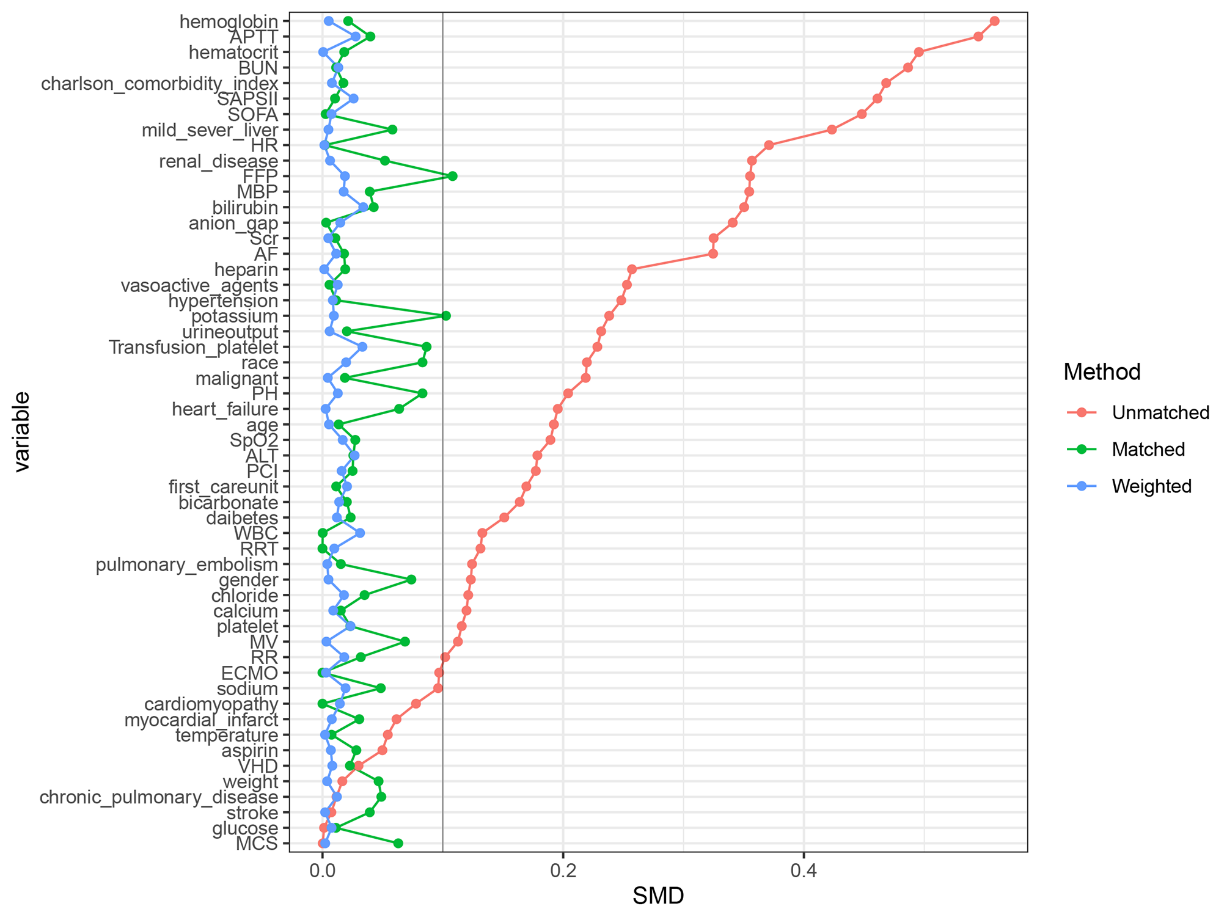
**Supplementary Figure 1.** Standardized mean difference (SMD) of variables before and after propensity score matching and weighting in the original cohort.

**Supplementary Table 1.** The baseline characteristics between survivors and non-survivors at discharge in validation cohort.

| **Variables** | **All** | **Survivors** | **Non-survivors** | ***p* value** |
| --- | --- | --- | --- | --- |
| N | 1350 | 599 | 751 |  |
| Age, years | 65.00 (54.00-74.00) | 63.00 (52.00-72.00) | 67.00 (55.00-76.00) | <0.001 |
| Male, % | 806 (59.70%) | 385 (64.27%) | 421 (56.06%) | 0.002 |
| Race, % |  |  |  | 0.728 |
| White | 987 (73.11%) | 438 (73.12%) | 549 (73.10%) |  |
| Black | 192 (14.22%) | 83 (13.86%) | 109 (14.51%) |  |
| Asian | 25 (1.85%) | 9 (1.50%) | 16 (2.13%) |  |
| Other | 146 (10.81%) | 69 (11.52%) | 77 (10.25%) |  |
| First care unit |  |  |  | 0.030 |
| CCU | 391 (28.96%) | 175 (29.22%) | 216 (28.76%) |  |
| MICU | 761 (56.37%) | 320 (53.42%) | 441 (58.72%) |  |
| Other | 198 (14.67%) | 104 (17.36%) | 94 (12.52%) |  |
| **Vital signs** |  |  |  |  |
| HR, beats/minute | 91.89 (75.25-106.00) | 91.00 (75.00-105.00) | 91.89 (76.00-106.50) | 0.299 |
| RR, times/minute | 20.00 (16.25-24.00) | 20.00 (16.00-23.00) | 20.85 (17.00-24.00) | 0.009 |
| MBP, mmHg | 83.37 (69.67-93.33) | 83.37 (73.00-95.00) | 83.00 (67.67-92.67) | <0.001 |
| Temperature, ℃ | 36.10 (35.00-36.70) | 36.40 (35.73-36.90) | 35.73 (34.40-36.50) | <0.001 |
| SpO2, % | 97.00 (95.62-100.00) | 98.00 (95.62-100.00) | 97.00 (95.62-100.00) | 0.099 |
| Weight, kg | 84.05 (70.00-100.00) | 85.00 (70.97-100.00) | 83.70 (69.35-100.00) | 0.429 |
| Urine amount, L | 1.36 (0.62-2.23) | 1.68 (0.92-2.66) | 1.12 (0.52-1.74) | <0.001 |
| APACHE-IV | 105.51 (85.00-128.00) | 96.00 (69.50-111.00) | 117.00 (101.00-139.00) | <0.001 |

Supplementary Table 1. Continued.

| **Variables** | **All** | **Survivor** | **Non-survivors** | ***p* value** |
| --- | --- | --- | --- | --- |
| **Laboratory tests** |  |  |  |  |
| Hemoglobin, g/dL | 10.30 (8.60-12.40) | 10.60 (8.90-12.80) | 10.10 (8.30-12.10) | <0.001 |
| Platelet, K/µl | 166.00 (121.00-211.00) | 169.71 (131.00-219.50) | 159.00 (110.50-207.00) | 0.001 |
| WBC, K/µl | 10.10 (7.00-14.00) | 10.00 (7.04-13.30) | 10.20 (6.90-14.45) | 0.619 |
| Hematocrit | 32.00 (26.30-37.90) | 32.80 (27.25-38.25) | 31.30 (25.70-37.05) | 0.011 |
| Anion gap, mmol/L | 11.58 (9.00-13.00) | 11.00 (8.00-12.00) | 11.58 (9.00-14.00) | <0.001 |
| Bicarbonate, mmol/L | 18.65 (15.00-22.00) | 19.00 (17.00-23.00) | 18.00 (14.00-21.00) | <0.001 |
| Calcium, mmol/L | 8.02 (7.50-8.60) | 8.10 (7.60-8.70) | 8.00 (7.30-8.50) | <0.001 |
| Chloridion, mmol/L | 101.00 (97.00-105.00) | 102.00 (98.00-105.00) | 101.00 (96.00-105.00) | 0.019 |
| Sodium, mmol/L | 137.00 (133.00-140.00) | 137.00 (134.00-139.00) | 137.00 (133.00-140.00) | 0.587 |
| Potassium, mmol/L | 3.50 (3.10-4.00) | 3.60 (3.10-3.90) | 3.50 (3.10-4.00) | 0.722 |
| Creatinine, mg/dl | 1.20 (0.84-1.90) | 1.07 (0.76-1.61) | 1.31 (0.90-2.10) | <0.001 |
| BUN, mg/dl | 21.00 (14.00-32.75) | 18.00 (12.00-28.00) | 22.00 (15.00-36.50) | <0.001 |
| Glucose, mg/dl | 117.00 (93.00-146.75) | 117.00 (94.00-143.00) | 117.00 (92.00-154.50) | 0.415 |
| ALT | 207.00 (53.00-331.02) | 154.00 (46.00-331.02) | 241.00 (59.00-331.02) | <0.001 |
| Bilirubin | 0.60 (0.40-0.87) | 0.60 (0.40-0.87) | 0.60 (0.40-0.90) | 0.757 |
| INR | 1.40 (1.14-1.95) | 1.26 (1.10-1.60) | 1.50 (1.20-2.20) | <0.001 |
| APTT | 34.00 (28.00-37.40) | 33.00 (28.00-36.01) | 34.80 (28.50-40.00) | 0.772 |
| **Comorbidities, n (%)** |  |  |  |  |
| Hypertension | 112 (8.30%) | 62 (10.35%) | 50 (6.66%) | 0.015 |
| Diabetes mellitus | 151 (11.19%) | 78 (13.02%) | 73 (9.72%) | 0.056 |

Supplementary Table 1. Continued.

| **Variables** | **All** | **Survivors** | **Non-survivors** | ***p* value** |
| --- | --- | --- | --- | --- |
| **Comorbidities, n (%)** |  |  |  |  |
| Heart failure | 114 (8.44%) | 61 (10.18%) | 53 (7.06%) | 0.04 |
| Atrial fibrillation | 132 (9.78%) | 64 (10.68%) | 68 (9.05%) | 0.316 |
| AMI | 144 (10.67%) | 81 (13.52%) | 63 (8.39%) | 0.002 |
| VHD | 13 (0.96%) | 9 (1.50%) | 4 (0.53%) | 0.07 |
| Cardiomyopathy | 48 (3.56%) | 24 (4.01%) | 24 (3.20%) | 0.424 |
| Pulmonary embolism | 31 (2.30%) | 11 (1.84%) | 20 (2.66%) | 0.314 |
| Pulmonary hypertension | 14 (1.04%) | 8 (1.34%) | 6 (0.80%) | 0.334 |
| COPD | 67 (4.96%) | 30 (5.01%) | 37 (4.93%) | 0.945 |
| Renal diseases | 88 (6.52%) | 43 (7.18%) | 45 (5.99%) | 0.38 |
| Liver diseases | 80 (5.93%) | 27 (4.51%) | 53 (7.06%) | 0.049 |
| Stroke | 36 (2.67%) | 17 (2.84%) | 19 (2.53%) | 0.727 |
| Malignancy | 9 (0.67%) | 3 (0.50%) | 6 (0.80%) | 0.739 |
| **Therapies, n (%)** |  |  |  |  |
| Mechanical ventilation | 1094 (81.04%) | 463 (77.30%) | 631 (84.02%) | 0.002 |
| RRT | 84 (6.22%) | 33 (5.51%) | 51 (6.79%) | 0.333 |
| Vasopressor | 699 (51.78%) | 247 (41.24%) | 452 (60.19%) | <0.001 |
| PCI | 115 (8.52%) | 66 (11.02%) | 49 (6.52%) | 0.003 |
| Aspirin | 50 (3.70%) | 33 (5.51%) | 17 (2.26%) | 0.002 |
| Heparin | 18 (1.33%) | 9 (1.50%) | 9 (1.20%) | 0.628 |

Supplementary Table 1. Continued.

| **Variables** | **All** | **Survivors** | **Non-survivors** | ***p* value** |
| --- | --- | --- | --- | --- |
| **Therapies, n (%)** |  |  |  |  |
| Transfusion of FFP | 37 (2.74%) | 12 (2.00%) | 25 (3.33%) | 0.138 |
| Transfusion of platelet | 11 (0.81%) | 3 (0.50%) | 8 (1.07%) | 0.252 |

CCU, cardiac care unit; MICU, medical intensive care unit. HR, heart rate; RR, respiratory rate; MBP, mean blood pressure; WBC, white blood cell; BUN, blood urea nitrogen; ALT, alanine transaminase; INR, international normalized ratio; APTT, activated partial thromboplastin time; APACHE-IV, acute physiology and chronic health evaluation IV; AMI, acute myocardial infarction; VHD, valvular heart disease; COPD, chronic obstructive pulmonary disease; RRT, renal replacement therapy; PCI, percutaneous coronary intervention; FFP, fresh frozen plasma.

**Supplementary Table 2.** Performance evaluation of INR, SOFA, and SAPSII in predicting the 1-year all-cause mortality of post-CA patients.

| **Variables** | **Cut-off** | **Area under curve** | **Confidence interval (95%)** | **Sensitivity** | **Specificity** |
| --- | --- | --- | --- | --- | --- |
| INR | 1.2 | 0.647 | 0.617-0.677 | 0.515 | 0.713 |
| SOFA | 6 | 0.632 | 0.601,0.663 | 0.746 | 0.448 |
| SAPSII | 39 | 0.684 | 0.654,0.714 | 0.752 | 0.528 |

CA, cardiac arrest; INR, international normalized ratio; SOFA, the sequential organ failure assessment; SAPSII, the simplified acute physiology score II.
